# A novel method for detection of HBVcccDNA in hepatocytes using rolling circle amplification combined with in situ PCR

**DOI:** 10.1186/s12879-014-0608-y

**Published:** 2014-12-03

**Authors:** Yanwei Zhong, Shuangye Hu, Chen Xu, Yulai Zhao, Dongping Xu, Yanqing Zhao, Jingmin Zhao, Zhibin Li, Xiuchang Zhang, Hongfei Zhang, Jin Li

**Affiliations:** Institute of Infectious Diseases, Beijing 302 Hospital, Beijing, China; He Bei North University, Zhangjiakou, China

**Keywords:** Covalently closed circular DNA, Hepatitis B virus, In situ PCR, Rolling circle amplification

## Abstract

**Background:**

Intrahepatic hepatitis B virus (HBV) covalently closed circular DNA (cccDNA) is the original template for HBV replication. The persistence of cccDNA is responsible for the recurrence of HBV infection. The detection of cccDNA can help the development of new antiviral drugs against HBV replication links, and reduce the resistance and recurrence as well as to discover extrahepatic HBV infection. In situ polymerase chain reaction (IS-PCR) can be used to determine the distribution and localization of cccDNA in liver tissues, but it is hampered by its low sensitivity and specificity. We developed a novel method to detect HBV cccDNA using rolling circle amplification (RCA) combined with IS-PCR.

**Methods:**

Biopsy liver tissues were obtained from 26 patients with HBV infection, including 10 chronic hepatitis B (CHB), 6 liver cirrhosis (LC) and 10 hepatocellular carcinoma (HCC) patients. Four pairs of primers were designed to mediating RCA for the first round amplification of HBV cccDNA specifically. The liver tissue sections from patients were treated by plasmid-safe ATP-dependent DNase (PSAD) prior to RCA. After RCA, HBV cccDNA was further amplified by a pair of selective primers labeled digoxigenin that target the gap region between the two direct repeat regions (DR1 and DR2) of the virus via IS-PCR.

**Results:**

HBVcccDNA was expressed and located in hepatocyte nucleus in 19 patients (73.07%). Compared with the IS-PCR, the introduction of RCA increase the limit of detection. RCA combined with IS-PCR yielded strong positive signals in HCC liver tissue in spite of low copy number cccDNA (2 copies of target sequence per cell), meanwhile, no positive signal was detected via negative control.

**Conclusions:**

RCA combined with IS-PCR is an effective and practicable method which could detect the presence of low copy number of cccDNA sensitively and specifically, and reflect the relationship between cccDNA expression level and liver tissue pathological characteristics.

**Electronic supplementary material:**

The online version of this article (doi:10.1186/s12879-014-0608-y) contains supplementary material, which is available to authorized users.

## Background

Chronic hepatitis B virus (HBV) infection remains a serious health problem that currently affects over 350 million people in the world and 93 million people in China [1]-[3]. It is estimated that 500 000 to 1 000 000 people die each year from HBV-related liver disease, such as liver cirrhosis (LC), hepatic decompensation, and hepatocellular carcinoma (HCC) [4]. Treatment of chronic hepatitis B (CHB) is aimed at suppressing viral replication to the lowest possible level, and thereby to halt the progression of liver disease and prevent the onset of complications [5]. However, it has always been hampered by the high relapse rate after discontinuation of the treatment. One crucial step within the HBV life cycle is the formation of covalently closed circular DNA (cccDNA), which serves as original template for viral replication with approximately 5–50 cccDNA molecules per infected hepatocyte [6]. Intrahepatic HBV cccDNA is responsible for the persistence of the disease in hepatocytes and it has been a key indicator to evaluate anti-HBV therapeutic efficacy [7],[8]. In addition, the HBV cccDNA level is considered to be valuable for studying HBV recurrence after liver transplantation and extrahepatic HBV infection [9],[10].

The development of highly selective real-time polymerase chain reaction (PCR) assays has provided sensitive tools to investigate the replicate activity and the effectiveness of antiviral therapy in infected patients by detection and quantification of the cccDNA in human liver biopsies [11]-[13]. However, the problem of nonspecific amplification of relaxed circular DNA (rcDNA) is a major obstacle in the development of an accurate cccDNA quantification assay. In a previous study, we improved and developed a quantification by introducing rolling circle amplification (RCA) steps combined with plasmid-safe ATP-dependent DNase (PSAD) digestion and real-time PCR, which offered better resolution [14]. However, these quantitative analysis methods could not reveal the distribution and localization of HBV cccDNA in liver tissues and further analyze the relationship between cccDNA and pathological characteristics.

Therefore, we developed a new sensitive and specific method for the locating detection of HBVcccDNA in sections from formalin fixed paraffin-embedded (FFPE) liver tissues by using RCA combined with in situ polymerase chain reaction (IS-PCR).

## Methods

### Ethics statement

This study was approved by the ethics committee of Beijing 302 Hospital. In this study, all samples were the residuary tissues of pathological examination, limited to in vitro studies, and didn’t have any contact and harm to patients, the institutional review board has waived the need for written informed consent from the participants.

### Study subjects

FFPE liver biopsy tissues (from the residuary tissues of pathological examination) were obtained from 26 HBV infected patients (21 male, 5 female; median age 39.73 years), hospitalized in Beijing 302 Hospital during a period from July 2011 - July 2012. According to the imaging and histological examination of liver, the patients were grouped into 10 CHB patients, 6 LC patients and 10 HCC patients. The characteristics of the patients are shown in Table 1. None of the patients received anti-HBV agents or steroids within six months prior to sampling. Concurrence of HCV, HDV, HIV infection, autoimmune liver disease and alcoholic liver disease was excluded for all enrolled individuals. FFPE liver biopsy tissues from four subjects without HBV infection (including two patients with HCV, one liver transplant from a healthy donor) and one of mice which transgenic complete HBV genomes) were collected as negative control.

**Table 1:** **Patient profiles and results of the present study**

Patient No.	Age (year)	Gender	Serum ALT (IU/L)	HBs Ag	HBe Ag	Serum HBV DNA (IU/mL)	PSAD + RCA + IS-PCR detect HBVcccDNA	Note
CHB								
1	0-10	M	89	+	+	1.33× 10^7^	+	Figure 2
2	21-30	M	68	+	+	1.46× 10^2^	+	
3	21-30	M	22	+	+	3.595× 10^2^	+	
4	21-30	M	197	+	-	7.51× 10^4^	+	
5	11-20	M	21	+	+	3.54× 10^8^	+	
6	11-20	M	13	+	+	1.51× 10^3^	-	
7	50-60	M	45	+	-	1.2× 10^5^	-	
8	50-60	M	263	+	+	2.73× 10^5^	+	
9	20-30	F	54	+	-	3.03× 10^4^	-	
10	20-30	F	381	+	+	4.75× 10^7^	+	
LC								
1	40-50	M	20	+	-	5.13× 10^7^	+	Figure 3
2	40-50	M	58	+	-	1.509× 10^4^	+	
3	40-50	M	33	+	-	1.17× 10^3^	+	
4	50-60	M	25	+	+	4.542× 10^3^	+	
5	50-60	M	136	+	-	3.08× 10^2^	-	
6	40-50	F	44	+	+	U.D.	-	
HCC								
1	30-40	M	55	+	-	U.D.	+	Figure 4
2	40-50	M	47	+	-	U.D.	+	
3	40-50	M	12	+	-	2.829× 10^2^	-	
4	40-50	M	44	+	-	2.446× 10^6^	+	
5	50-60	F	25	+	+	2.182× 10^4^	+	
6	50-60	M	14	+	-	1.11× 10^3^	+	
7	60-70	M	88	+	+	1.79× 10^3^	+	
8	60-70	M	17	-	-	U.D.	+	
9	40-50	F	35	+	-	4.464× 10^3^	-	
10	30-40	M	206	+	-	4.44× 10	+	

M, male; F, female; U.D., undetected; HBV, hepatitis B virus; ALT, alanine aminotransferase; HBsAg, hepatitis B surface antigen; HBeAg, hepatitis B e antigen; PSAD, plasmid-safe ATP-dependent DNase; RCA, rolling circle amplification; IS-PCR, situ polymerase chain reaction; CHB, chronic hepatitis B; LC, liver cirrhosis; HCC, hepatocellular carcinoma.

### Serological markers and quantification of HBV DNA and cccDNA

Serum ALT, HBeAg/anti-HBe and other serological markers were routinely measured or detected in the Central Clinical Laboratory of the Beijing 302 Hospital. Serum HBV DNA level was determined by real -time quantitative PCR kit (Fosun Pharmaceutical Co., Ltd., Shanghai, China) with a lower detection limit of 100 IU/ml (≈500 copies/ml). Quantification of intrahepatic HBV cccDNA using rolling circle amplification in combination with real-time PCR according to the literature [14]. Intrahepatic HBV cccDNA levels were normalized by the amount of human genomic (hg)-beta-actin in the samples. Cell numbers were calculated based on an estimation of 6.667 pg/hgDNA per cell.

### Sample preparation

The liver tissue samples were fixed in 10% buffered formalin (pH 7.4) for 24 hrs, embedded in paraffin, cut into 5μm sections, and mounted on 0.05% poly-L-Lysine glass slides, followed by incubation at 60°C for 2 hrs. Tissue sections (5μm) were first stained with hematoxylin and eosin (HE) and studied to confirm the adequacy of the biopsy.

### Deparaffinization and digestion

The sections were deparaffinized with two pre-warmed xylene washes followed by 95%, 75%, and 50% ethanol rinses. The sample slides were dried at room temperature after washing in distilled water. After deparaffinizing by the normal method, the tissue sections on the glass slides were digested with proteinase K (Boster Biotech, Wuhan, China) 50 μg/ml at 37°C for 10 min in a humidified chamber and then the sections were rinsed with phosphate-buffer saline (PBS). PSAD (Epicentre, Madison, Wisconsin, USA) was used to digest HBV rcDNA, replicate double-stranded DNA (dsDNA) and single-stranded DNA (ssDNA). The reaction mixture contained 3U PSAD, 1.0 mmol/l ATP and 1 μl 10 × reaction buffers, with doubly distilled water (ddH_2_O) to final volume of 15.5 μl. The digestion was carried out at 37°C for 30 min and inactivated at 70°C for 30 min. Endogenous alkaline phosphatase (AP) in the liver tissues was removed byusing 20% acetic acid (in order to increase the permeability of cell membrane) for 10 min at room temperature. Subsequently, the sections were fixed in 4% formaldehydum polymerisatum, rinsed with distilled water, dehydrated by 75% ethanol, 95% ethanol and 100% ethanol, and air dried.

### RCA treatment

Four pairs of primers were designed for mediating RCA for the first amplification round of HBV cccDNA specifically (Table 2). In order to increase the efficiency of amplification, RCA was divided into two steps: First-step, 14 μl primer combining reaction mixture containing primers at a operative solutionconcentration of 0.5 μmol/l each , 2 μl reaction buffer and 11 μl ddH_2_O were loaded onto the tissue sections on glass slides and sealed with rubber cement, followed by heating at 95°C for 6 min, and then placed on ice for 15 min. Second-step the RCA amplification was as follows: 23 μl of reaction mixture was added to tissue slides containing primers at a operative solution concentration of 0.5 μmol/l each, 10 mg/ml bovine serum albumin (BSA), 0.32 mmol/l of dNTP, 20 U of the Phi29 DNA polymerase (New England Biolabs, Worcester, MA), and 2 μl reaction buffer. Reaction was carried out overnight for 16 hrs at 30°C. Then Phi29 DNA polymerase was inactivated at 70°C for10 min. Subsequently, the sections were fixed in 4% formaldehydum polymerisatum, rinsed with distilled water, dehydrated in a graded series of alcohol and air dried.

**Table 2:** **Oligonucleotide sequences of primers used in this study**

Name	Sequence (5′→3′)	Nucleotide position	Polarity
Primers for rolling circle amplification (RCA)			
RCA1	AATCCTCACAATA^*^C^*^C	226-240	Sense
RCA2	ACCTATTCTCCTC^*^C^*^C	1744-1758	Antisense
RCA3	CCTATGGGAGTGG^*^G^*^C	637-651	Sense
RCA4	CCTTTGTCCAAGG^*^G^*^C	2675-2689	Antisense
RCA5	ATGCAACTTTTTC^*^A^*^C	1814-1828	Sense
RCA6	CTAGCAGAGCTTG^*^G^*^T	15-29	Antisense
RCA7	TAGAAGAAGAACT^*^C^*^C	2368-2382	Sense
RCA8	GGGCCCACATATT^*^G^*^T	2585-2599	Antisense
Primers for in situ PCR (IS-PCR)			
Pup58	CCCCGTCTGTGCCTTCTC	1547- 1564	Sense
Pdown84	CAGCTTGGAGGCTTGAACAGT	1859-1879	Antisense

*Indicates primer phosphorothioate modifications so as to prevent degradation by nucleases.

### IS-PCR and immunohistochemical staining for detecting HBV cccDNA

HBV cccDNA was further amplified with IS-PCR mediated by a pair of cccDNA-selective primers labeled with digoxigenin at 5′-terminal, that targets the gap region between the two direct repeat regions (DR1 and DR2) of the viral genome (Table 2) [14]. 23.5 μl reaction solution containing a operative solutionconcentration of 0.32 mmol/L dNTP, 10 mg/ml BSA, 3.5 μmol/l MgCl_2_, 5 μl of Taq polymerase PCR buffer, 5 μl of ddH_2_O, 0.2 μmol/l of selective primers, and 10 U Taq polymerase (TaKaRa Biotechnology, Dalian, China) was added to the tissue slides and sealed with rubber cement. The slides were encapsulated with silver paper and placed in the thermal cycle system (ThermoPX2, USA) at 95°C for 3 min followed by 30 cycles of 95°C for 1 min, 55°C for 90 s, 72°C for 90 s and a final extension at 72°C for 10 min. Immediately after the PCR, the slides were fixed in 4% paraformaldehyde for 10 min, washed in Tris–HCl buffer saline (TBS) for 3 min, rinsed with distilled water for 3 min, dehydrated in a graded series of alcohol and air dried.

After IS-PCR, sample slides were washed with 0.1% Triton X-100 in Tris–HCl buffer saline (TBS-T) for 10 min and TBS for 3 min at room temperature. After incubated in blocking reagent 3% BSA at 37°C temperature for 10 min, the slides were covered with 100 μl Alkaline phosphatase, conjugated Monoclonal Anti-Digoxin (SIGMA, Saint Louis, Missouri, USA) and 1 than 30 dilution with TBS at 37°C for 120 min. After this reaction, the slides were washed twice (3 min each) with TBS. The slides were incubated in 30 μl dye solution (338 μg/ml nitroblue tetrazolium chloride NBT, 175 μg/ml 5-bromo-4-chloro-3-indolyl-phosphate 4-toluidine salt BCIP) at 37°C in the dark. After sufficient color development, they were washed with de-ionized water for three times and then nuclear fast red re-stained. Finally, the slides were sealed by using neutral balsam for microscopic examination.

### Evaluation of sensitivity, repeatability and specificity of the assay

To evaluate this method’s sensitivity, we chose a liver tissue with low copy number (2 copies cccDNA per cell) from a HCC patient as template, which the quantification of intrahepatic HBV cccDNA according to our previous study [14]. The repeatability was evaluated by detecting the serial sections which were cut from same sample in inter-assay and intra-assay. Intra-assay reproducibility was assessed by repeating cccDNA detection of FFPE liver biopsy samples selected from three patients randomly from CHB、LC、HCC respectively for three times in a single run. Inter-assay variation was evaluated by detecting the three patients’ samples mentioned above in three independent experiments. The specificity was assessed by examining FFPE liver biopsy tissue taken from the patients with HCV, from healthy adults and from transgenic mice. In addition, FFPE liver biopsy tissue from the patient with HCC, which were amplified in situ omitting specific primer, labled primers, phi29 polymerase and Taq DNA polymerase respectively, instead of ddH_2_O, were also examined for evaluation of the specificity of the assay.

### Methodological comparison

Liver tissues from patients with CHB and HBV-related LC, HCC were detected using three different methods as follows:

1. Amplification of samples by IS-PCR (IS-PCR).
2. Treatment of samples with PSAD by IS-PCR (PSAD + IS-PCR).
3. Treatment of samples with PSAD followed by RCA prior to IS-PCR (PSAD + RCA + IS-PCR).

### Nucleotide sequence accession numbers

The sequences accession numbers of the HBV genomes in GenBank are EU939666, FJ386583, FJ386632 and FG386652.

## Results

### Localization of HBVcccDNA

19 of 26 (73.07%) samples from HBV-infected patients including 7 of 10 CHB, 4 of 6 LC and 8 of 10 HCC were found positive for cccDNA by in situ amplification using PSAD + RCA + IS-PCR (Table 1). Liver cells with positive staining were scattered in tissues with clustering, and the signals compartment in the nuclear, staining blue or blue-violet. Interestingly, strong positive signals could be observed in the liver tissue from the patients with HCC despite of low copy number cccDNA. The detection threshold is 2 copies of target sequence per cell.

### Intra-assay and inter-assay variability experiments

The positive expression intensity and distribution of replicate test were essentially consistent. Repeated experiments showed that this method shows good stability and reproducibility.

### Control study

**Figure 1:**
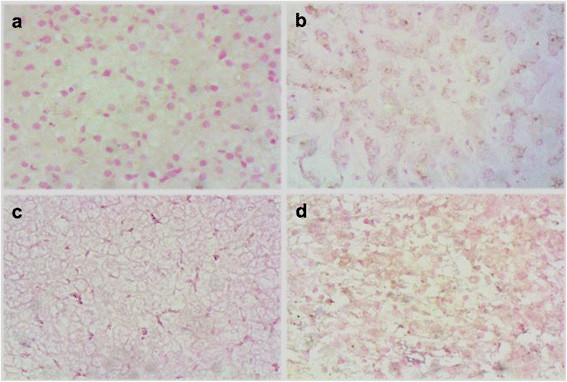
**IS-PCR and immunohistochemical staining for detecting HBV cccDNA in FFPE liver biopsies.** No positive signal was detected in liver tissue samples from HCV patient **(a)**, healthy adults **(b)**, transgenic mice **(c)** and HCC patients without specific primers **(d)** by PSAD + RCA + IS-PCR. Original Magnifications × 200.

The specificity of PSAD + RCA + IS-PCR method was examined in a serious of control experiments. No positive signals were detected when the primers, phi29 polymerase or Taq DNA polymerase were omitted, neither in the liver specimens from the patients with HCV, healthy adults and transgenic mice (Figure 1).

### Methodological comparison

**Figure 2:**
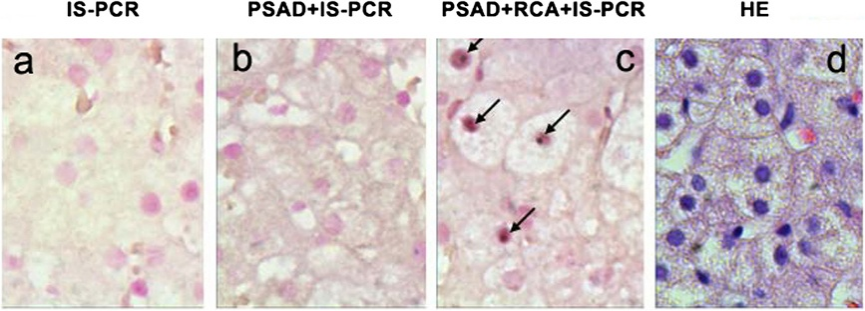
**IS-PCR and immunohistochemical and HE staining for detecting HBV cccDNA in FFPE liver biopsies of CHB patient.** HBVcccDNA were detected in the liver tissues by PSAD + RCA + IS-PCR **(c)**, whereas no positive signal was detected in the tissue sections that were subjected to IS-PCR **(a)** and PSAD + IS-PCR **(b)**. **(d)** hematoxylin-eosin staining of FFPE liver biopsies of CHB patient. The arrow in the images shows HBVcccDNA positive signals in the hepatocyte nuclei. Original magnifications ×400.

**Figure 3:**
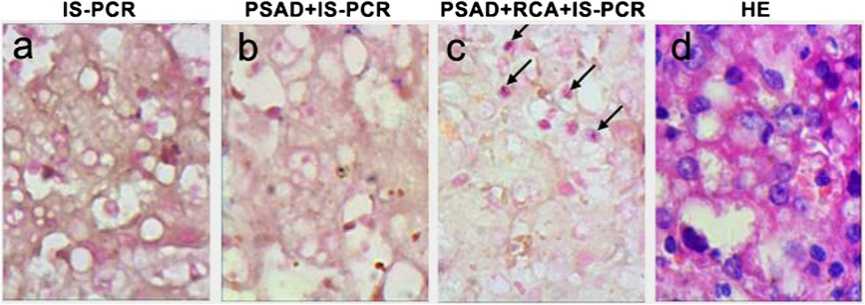
**IS-PCR and immunohistochemical and HE staining for detecting HBV cccDNA in FFPE liver biopsies of LC patient.** HBV cccDNA were detected in the liver tissues by PSAD + RCA + IS-PCR **(c)**, whereas no positive signal was detected in the tissue sections that were subjected to IS-PCR **(a)** and PSAD + IS-PCR **(b)**. **(d)** hematoxylin-eosin staining of FFPE liver biopsies of LC patient. The arrow in the images shows HBVcccDNA positive signals in the hepatocyte nuclei. Original magnifications ×400.

**Figure 4:**
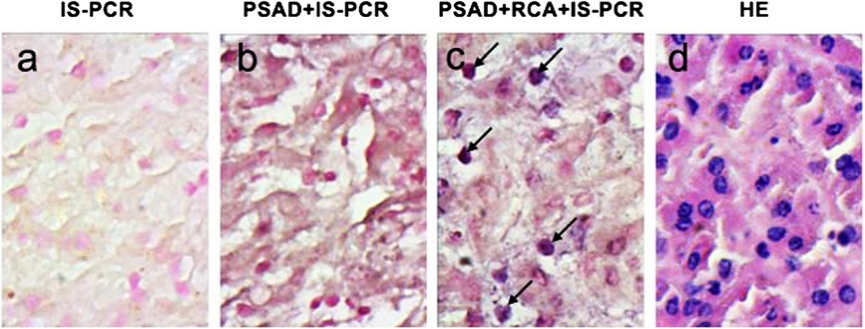
**IS-PCR and immunohistochemical and HE staining for detecting HBV cccDNA in FFPE liver biopsies of HCC patient.** HBVcccDNA were detected in the liver tissues by PSAD + RCA + IS-PCR **(c)**, whereas no positive signal was detected in the tissue sections that were subjected to IS-PCR **(a)** and PSAD + IS-PCR **(b)**. **(d)** hematoxylin-eosin staining of FFPE liver biopsies of HCC patient. The arrow in the images shows HBVcccDNA positive signals in the hepatocyte nuclei. Original magnifications ×400.

Compared with IS-PCR methods, PSAD + RCA + IS-PCR increased the sensitivity and specificity of HBV cccDNA detection. HBVcccDNA were detected in the liver tissues from patients with CHB (Figure 2), LC (Figure 3) and HCC (Figure 4) by PSAD + RCA + IS-PCR, whereas no positive signal was detected in the consecutive tissue sections that were subjected to IS-PCR and PSAD + IS-PCR.

## Discussion

HBV cccDNA plays a key role in the life cycle of the HBV and permits the persistence of infection. Studies have reported a development or improvement in the methodology to quantify viral cccDNA more specifically and sensitively [11],[15]-[17]. Using this method, we developed a quantification of HBVcccDNA in FFPE liver tissue using RCA combined with real-time PCR and examined 130 samples from the patients with CHB in our previous studies [14]. In order to observe distribution and localization of HBV cccDNA in liver tissues and further analyze the relationship between cccDNA and pathological characteristics, we developed a highly specific and sensitive method to detect the HBV cccDNA in liver tissues via RCA combined with IS-PCR.

RCA is a novel isothermal DNA amplification method with an amplification mechanism quite different from conventional PCR. This method uses a specially designed circular probe (C-probe) in which the 3′ and 5′ ends are brought together in juxtaposition by hybridization to a target. The two ends are then covalently linked by a T4 DNA ligase in a target-dependent manner, producing a closed DNA circle. In the presence of an excess of primers (forward and reverse primers), a DNA polymerase extends the bound forward primer along the C-probe and displaces the downstream strand, generating a multimeric single-stranded DNA (ssDNA), analogous to the “rolling circle” replication of bacteriophages in vivo. This multimeric ssDNA then serves as a template for multiple reverse primers to hybridize, extend, and displace downstream DNA, generating a large ramified (branching) DNA complex. This ramification process continues until all ssDNAs become double-stranded, resulting in an exponential amplification that distinguishes itself from the previously described nonexponential rolling circle amplification [18]. The major advantage of RCA is its efficiency and fidelity. It exhibits less amplification bias and greater yield, product length, and fidelity than conventional PCR [19]. In 2008, Margeridon et al. [20] first applied RCA for qualitatively probing HBV cccDNA. We also applied RCA to analyze drug resistant mutations of HBV cccDNA in patients with chronic hepatitis B [21]. RCA relies on the properties of the phi29 DNA polymerase that possesses a strong 3 exonuclease (proofreading) activity, is able to polymerize more than 70,000 nucleotides without detaching from the template, and that can displace previously elongated strands [20]. Phi29 DNA polymerase used in RCA can selectively amplify circular template molecule [22], it minimizes the impact of integrated HBV DNA and obviously improves the sensitivity and specificity of HBV cccDNA assay. In the present study, a more efficient amplification was operated by adding 4 pairs of RCA primers targeting multiple binding sites. Therefore, RCA were optimized to improve their sensitivity without impairing the specificity.

IS-PCR, which is the combination of PCR and ISH, was first described by Haase et al. in 1990 [23]. IS-PCR has the advantages of both PCR and ISH. It has been adapted to in situ amplification of viral genomes or their replicate intermediates in liver tissue sections [24],[25] and can localize a single gene copy at the individual cell level [26]-[28]. But the sensitivity and specificity remain major challenges to the application.

In practice, IS-PCR is technically difficult, especially when using paraffin-embedded tissue sections, because there is no standard protocol for optimal detection. In fact, in this method the diffusion of the amplified products from the target cells is a major problem. The factors that influence diffusion include excessive protease digestion [26],[29] and the use of an excessive number of PCR amplification cycles [30],[31]. The key step in the IS-PCR is a controlled proteinase K pretreatment. Optimal proteinase treatment permeates sections sufficiently to permit entry of PCR reagents into cells without leakage of the PCR products or loss of tissue morphology. So, the concentration of proteinase K and the treatment time were adjusted to optimize permeability of membranes and the release of protein-nucleic acid cross-linking, while avoiding over digestivity. To minimize diffusion of the PCR products, we chose the optimal proteinase K concentration (50 μg/ml for paraffin sections) for 10 min, which produced a positive sharp in situ PCR signal. We also limited the number of PCR cycles, which was important for eliminating the background staining, as too many cycles resulted in high background staining and loss of tissue morphology. Finally, we fixed the liver tissue sections in 4% paraformaldehyde immediately after PCR amplification: this step is essential to avoid diffusion of PCR products into neighboring cells.

In this study, First of all, we used PSAD to digest linearization of the rcDNA. PSAD pretreatment proved to be necessary, without this step, there was an obvious increase of amplicons amount, which was reasonably coming from the nonspecific amplification. But the impact of integrated HBV DNA could not be excluded. To solve this problem, RCA in situ optimally performed at 30°C for 16 hrs. It can minimize the impact of integrated HBV DNA and obviously improve the specificity of HBV cccDNA assay. Finally, the slides were placed in an IS-PCR system and amplification reaction was performed as described in materials and methods. By means of this method, we obtained clear and reproducible patterns of distribution or localization of the HBV cccDNA in the liver tissue sections. Our results showed that HBV cccDNA were detected in a large number of hepatocytes in tissue sections from HBV-infected patients. Positive signals were observed in the nucleus despite of low copy number cccDNA (2 copies of target sequence per cell). These data indicate that the method is specific and sensitive for the detection of HBV cccDNA.

## Conclusions

RCA combined with IS-PCR could sensitively and specifically detect HBV cccDNA in infected hepatocytes, and visualize the tissue staining patterns. Distribution and localization of HBV cccDNA in liver tissues could be made visible to the unaided eye under a light microscope, which can reflect the relationship between cccDNA expression level and liver tissue pathological characteristics so as to evaluate anti-HBV therapeutic efficacy. Our assay also may provide a powerful tool for discovering the existence of extrahepatic cccDNA.

## Authors’ contributions

YWZ, SYH designed and drafted the manuscript. YWZ, SYH, CX, YLZ YQZ, ZBL performed experiments. YWZ, SYH, CX, DPX, JMZ, XCZ, HFZ, JL analyzed data and discussed results. All authors read and approved the final manuscript.

## Authors’ original submitted files for images

Below are the links to the authors’ original submitted files for images. Authors’ original file for figure 1 Authors’ original file for figure 2 Authors’ original file for figure 3 Authors’ original file for figure 4
