# Supplementary figures and images for: A novel method for detection of HBVcccDNA in hepatocytes using rolling circle amplification combined with in situ PCR
Source: BMC Infect Dis. 2014 Dec 3;14:608. doi: 10.1186/s12879-014-0608-y (PMC4264245; doi:10.1186/s12879-014-0608-y)

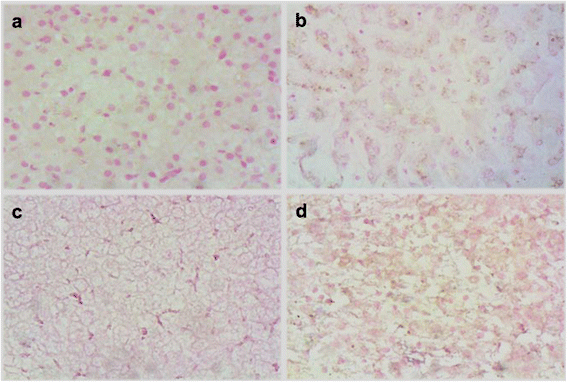

Supplement: Supplementary file 1 — Authors’ original file for figure 1 [file 12879_2014_608_MOESM1_ESM.gif]

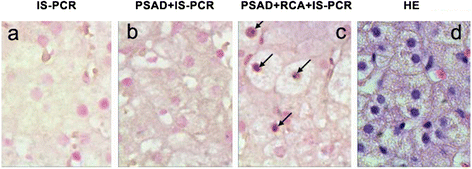

Supplement: Supplementary file 2 — Authors’ original file for figure 2 [file 12879_2014_608_MOESM2_ESM.gif]

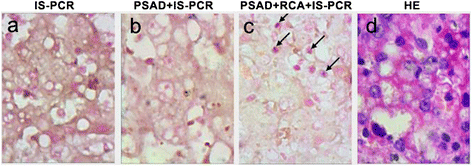

Supplement: Supplementary file 3 — Authors’ original file for figure 3 [file 12879_2014_608_MOESM3_ESM.gif]

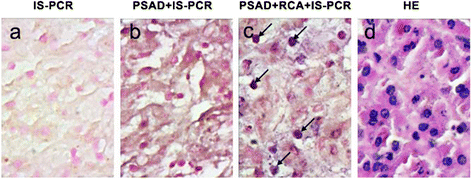

Supplement: Supplementary file 4 — Authors’ original file for figure 4 [file 12879_2014_608_MOESM4_ESM.gif]
